# Robot-assisted partial nephrectomy: Can retroperitoneal approach suit for renal tumors of all locations?—A large retrospective cohort study

**DOI:** 10.1186/s12894-022-01128-y

**Published:** 2022-12-10

**Authors:** Xiangjun Lyu, Zhuo Jia, Liyan Ao, Changhao Ren, Yangyang Wu, Yunlai Xu, Ke Chen, Yu Gao, Baojun Wang, Xin Ma, Xu Zhang

**Affiliations:** Department of Urology, The Third Medical Center, Chinese PLA General Hospital, 28 Fu Xing Road, Haidian District, Beijing, 100853 China

**Keywords:** Renal tumor, Retroperitoneal approach, Robot-assisted partial nephrectomy

## Abstract

**Background:**

This study aimed to explore the appropriate location of renal tumors for retroperitoneal approach.

**Materials and Methods:**

We retrospectively analyzed 1040 patients with renal tumor who were treated at our institution from Janurary 2015 to June 2020 and had underwent retroperitoneal robotic assisted-laparoscopic partial nephrectomy (rRAPN). Clinical features and postoperative outcomes were evaluated.

**Results:**

Patients with incomplete data were excluded, and we included 896 patients in total. The median tumor size was 3.0 (range: 0.8–10.0) cm. The median RENAL Nephrometry Score was 7 (range: 4–11), and the median PADUA Nephrometry Score was 8 (range: 6–14). The median surgical time was 120 min, and the median warm ischemia time was 18 min. The median estimated blood loss was 50 ml. The follow-up time was 20.2 (range: 12–69) months. The mean change of eGFR 1 year after operation was 14.6% ± 19.0% compared with preoperative estimated glomerular filtration rate (eGFR). When compared the tumor at different locations, as superior or inferior pole, anterior of posterior face of kidney, there were no significant differences of intra- and post-operative outcomes such as surgical time, warm ischemia time, estimated blood loss, removal time of drainage tube and catheter, postoperative feeding time and hospital stay, and changes of eGFR one year after surgery. We also compared tumors at special locations as endophytic or exophytic, anterior of posterior hilus of kidney, there were no significant differences in surgical time, warm ischemia time, estimated blood loss and changes of eGFR. There was no significant difference in intraoperative features and postoperative outcomes when tumor larger than 4 cm was located at different positions of kidney. Though the surgical time was longer when BMI ≥ 28 (132.6 min vs. 122.5 min, p = 0.004), no significant differences were observed in warm ischemia time, estimated blood loss, changes in eGFR. Twenty-seven patients (3.0%) had tumor progression, including 8 (0.9%) recurrence, 19 (2.1%) metastasis, and 9 (1.0%) death.

**Conclusion:**

Retroperitoneal approach for RAPN has confirmed acceptable intra- and postoperative outcomes and suits for renal tumors of all different locations. Large tumor size and obesity are not contraindications for rRAPN.

## Background

Nephron-sparing surgery is the recommended surgical treatment for T1a renal tumors [1] and a viable treatment option for select T1b and T2 renal tumors to preserve more normal renal parenchyma. [2] Robot-assisted partial nephrectomy (RAPN) for nephron sparing surgery is now widely used due to its safety and effectiveness in complex renal tumor and better outcomes than standard laparoscopy. [3–5] Transperitoneal (tRAPN) [6] and retroperitoneal (rRAPN) [7] approaches are standard methods for RAPN. The appropriate location of renal tumors for retroperitoneal approach still remains controversial. Some studies showed superiority for posterior tumors and challenges for anterior, medial, and inferior pole tumors, [8] while other studies showed no difference between rRAPN and tRAPN. [9, 10] This study is the largest cohort study in single center of patients that underwent rRAPN reported in literature to compare intraoperative data and postoperative outcomes of different locations of renal tumors.

## Materials and methods

We retrospectively studied 1040 patients with renal tumor, who were treated at our institution from January 2015 to June 2020, and had undergone retroperitoneal robotic assisted-laparoscopic partial nephrectomy. Patients with incomplete image and follow-up data were excluded. We included 896 patients in total. All the available clinical data were reviewed. Baseline clinical characteristics included age, gender, BMI, tumor size, tumor side, tumor location, RENAL Nephrometry Score (Radius, Exophytic, or endophytic properties, Nearness of tumor to the collecting system or sinus in millimeters, Anterior or posterior location to polar line), PADUA Nephrometry Score, surgical time, warm ischemia time, and estimated blood loss. Post-operative outcomes included perioperative outcomes (positive surgical margin rate, drainage tube and catheter removal time, feeding time, hospital stay, surgical complication), functional outcomes–changes in eGFR one year after operation, and oncological outcomes containing follow-up time, and disease progression (recurrence, metastasis, and death) rate.

### Statistical analysis

Clinical characteristics were compared by Wilcoxon test, chi-square, or Fisher exact test. Univariable analysis with log-rank test and multivariate analysis with cox proportional hazard regression model were used to estimate hazard ratios and 95% confidence intervals. Statistical analyses were performed using SPSS software version 23 (SPSS, Inc.), and two-tailed p < 0.05 were considered statistically significant.

## Results

The clinical characteristics of 896 patients are shown in Table 1. The male-to-female ratio was approximately 3:1. The median age was 52 (range: 14–86) years, and the median BMI was 25.8 kg m^2^. The median tumor size was 3.0 (range: 0.8–10.0) cm. For tumor location, 338 (37.7%) patients had tumor on superior pole of kidney, 252 (28.1%) had tumor on inferior pole, and the other 306 (34.2%) had tumor in the middle of kidney. As for face of tumor, 229 (25.6%) patients had tumor with anterior facing, 533 (59.5%) had posterior facing, and the other 134 (14.9%) had undefined result. The median RENAL Nephrometry Score was 7 (range: 4–11), and the median PADUA Nephrometry Score was 8 (range: 6–14). The median surgical time was 120 (range: 40–385) min, and the median time required for port insertion and docking was 23(range: 10–46) min. The warm ischemia time was 18 (range: 6–40) min and the median estimated blood loss was 50 (range: 5–2000) ml. Postoperative clinical features are shown in Table 2. The median removal times of drainage tube and catheter were 3 (range: 1–20) days and 2 (range: 1–13) days, respectively. The median postoperative feeding time and postoperative hospital stay were 2 (range: 1–10) days and 5 (range: 9–69) days, respectively. Surgical complications were evaluated by Clavien Dindo classification. The complication rate was 12.9% and rate of major postoperative complications was low (2.7% for Grade 2, 0.4% for Grade 3 and 0.1% for Grade 4). The follow-up time was 20.2 (range: 12–69) months. The mean changes of eGFR 1 year after operation was 14.6% ± 19.0% compared with preoperative eGFR. Twenty-seven patients (3.0%) had tumor progression, including 8 (0.9%) recurrence, 19 (2.1%) metastasis, and 9 (1.0%) death.

**Table 1:** Clinical features

Variables	n
n	896
*Gender*	
Male (%)	663 (74.0%)
Female	233 (26.0%)
Age (years)	52 (14–86)
BMI (kg.m2)	25.8 (17.0-50.8)
*Side (%)*	
Left	440 (49.0%)
Right	456 (51.0%)
Tumor size (cm)	3.0 (0.8–10.0)
*Superior or inferior location (%)*	
Superior pole	338 (37.7%)
Inferior pole	252 (28.1%)
Middle	306 (34.2%)
*Anterior or posterior location*	
Anterior (A)	229 (25.6%)
Posterior (B)	533 (59.5%)
Undefined (X)	134 (14.9%)
RENAL nephrometry score	7 (4–11)
PADUA nephrometry score	8 (6–14)
Surgical time (min)	120 (40–385)
Warm ischemia time (min)	18 (6–40)
Estimated blood loss (ml)	50 (5-2000)

**Table 2:** Postoperative clinical features

Variables	n
Drainage tube removal time (days)	3 (1–20)
Catheter removal time (days)	2 (1–13)
Postoperative feeding time (days)	2 (1–10)
Postoperative hospital stay (days)	5 (2–52)
Positive surgical margin (%)	4 (0.45%)
Complication (Clavien Dindo classification)	116 (12.9%)
Grade 1	87 (9.7%)
Grade 2	24 (2.7%)
Grade 3	4 (0.4%)
Grade 4	1 (0.1%)
Grade 5	0
Changes of eGFR (%)	14.6 ± 19.0
Follow up time (months)	20.2 (12–69)
Disease progression (%)	
Recurrence	8 (0.9%)
Metastasis	19 (2.1%)
Death	9 (1.0%)

Comparison of clinical characteristics of patients with tumor at superior or inferior pole and anterior or posterior face of kidney is shown in Table 3. All the features shown had no significant differences. The major postoperative complication rate was low and there was no statistical difference in comparison of different locations.

**Table 3:** Comparison of clinical characteristics of patients with tumor at superior or inferior pole and anterior or posterior face of kidney

Variables	Superior vs. Inferior	Anterior vs. Posterior
RENAL nephrometry score	6.2 vs. 6.1, p = 0.385	6.8 vs. 6.9, p = 0.347
PADUA nephrometry score	8.1 vs. 7.9, p = 0.145	8.7 vs. 8.7, p = 0.804
Surgical time (min)	126.6 vs. 123.6, p = 0.419	123.0 vs. 125.1, p = 0.562
Warm ischemia time (min)	18.9 vs. 19.8, p = 0.191	18.8 vs. 19.9, p = 0.111
Estimated blood loss (ml)	73.0 vs. 66.5, p = 0.262	71.5 vs. 63.5, p = 0.412
Complication (Clavien Dindo classification)		
Grade 1 (%)	9.5 vs. 9.9, p = 0.727	9.6 vs. 9.6, p = 0.904
≥Grade 2 (%)	3.3 vs. 2.8, p = 0.325	3.1 vs. 3.2, p = 0.937
Changes of eGFR (%)	16.4 vs. 14.4, p = 0.077	14.4 vs. 13.3, p = 0.462
Drainage tube removal time (days)	3.3 vs. 3.2, p = 0.627	3.2 vs. 3.4, p = 0.215
Catheter removal time (days)	2.4 vs. 2.5, p = 0.760	2.5 vs. 2.4, p = 0.790
Postoperative feeding time (days)	2.2 vs. 2.2, p = 0.932	2.2 vs. 2.1, p = 0.110
Postoperative hospital stay (days)	5.4 vs. 5.3, p = 0.978	5.6 vs. 5.4, p = 0.467

Table 4 shows the comparison of clinical characteristics of anterior and posterior renal tumor when located on superior, inferior, or middle of kidney. No significant differences were observed on patients that had undergone rRAPN when tumor was located at different positions.

**Table 4:** Comparison of clinical characteristics of anterior and posterior renal tumor located on superior, inferior, or middle of kidney

Variables	Superior pole	Inferior pole	Middle
n	87 vs. 206	70 vs. 159	82 vs. 168
RENAL nephrometry score	5.9 vs. 6.2, p = 0.096	6.1 vs. 6.0, p = 0.585	8.1 vs. 8.6, p = 0.057
PADUA nephrometry score	7.9 vs. 8.3, p = 0.086	8.3 vs. 7.8, p = 0.063	9.7 vs. 10.1, p = 0.102
Surgical time (min)	130.1 vs. 124.3, p = 0.326	122.7 vs. 123.7, p = 0.885	115.4 vs. 127.6, p = 0.048
Warm ischemia time (min)	18.4 vs. 18.8, p = 0.712	18.6 vs. 20.1, p = 0.281	19.1 vs. 21.1, p = 0.130
Estimated blood loss (ml)	89.9 vs. 64.4, p = 0.217	51.9 vs. 61.4, p = 0.429	66.0 vs. 64.6, p = 0.915
*Complication (Clavien Dindo classification)*			
Grade 1 (%)	10.3 vs. 8.7, p = 0.521	10.0 vs. 10.1, p = 0.836	7.3 vs. 10.1, p = 0.105
≥Grade 2 (%)	3.4 vs. 3.4, p = 0.928	2.9 vs. 2.5, p = 0.432	2.4 vs. 3.6, p = 0.220
Changes of eGFR (%)	18.9 vs. 15.6, p = 0.066	13.5 vs. 15.6, p = 0.304	16.2 vs. 16.3, p = 0.956
Drainage tube removal time (days)	3.2 vs. 3.3, p = 0.501	3.2 vs. 3.2, p = 0.871	3.3 vs. 3.7, p = 0.153
Catheter removal time (days)	2.5 vs. 2.3, p = 0.287	2.4 vs. 2.4, p = 0.905	2.5 vs. 2.6, p = 0.448
Postoperative feeding time (days)	2.4 vs. 2.1, p = 0.111	2.3 vs. 2.1, p = 0.244	2.0 vs. 2.1, p = 0.293
Postoperative hospital stay (days)	5.4 vs. 5.3, p = 0.720	5.0 vs. 5.5, p = 0.306	6.2 vs. 5.5, p = 0.188

We analysed the clinical characteristics of patients with tumor at special locations as endophytic tumor and hilus tumor of kidney. Table 5 shows the comparison of endophytic and exophytic renal tumor. There were no significant differences in tumor size (p = 0.842). RENAL and PADUA Nephrometry Score showed higher score in endophytic tumor group. No significant differences were observed in surgical time, warm ischemia time, estimated blood loss and changes in eGFR. Table 6 shows comparison of clinical features of anterior and posterior renal hilus tumor. All the characteristics showed no significant differences.

**Table 5:** Comparison of clinical characteristics of patients with exophytic tumor and endophytic tumor of kidney

Variables	Exophytic tumor vs. Endophytic tumor
n	668 vs. 223
Tumor size (cm)	3.0 vs. 3.1, p = 0.842
RENAL nephrometry score	6.4 vs. 8.6, p < 0.001
PADUA nephrometry score	8.0 vs. 10.9, p < 0.001
Surgical time (min)	125.5 vs. 122.8, p = 0.443
Warm ischemia time (min)	19.2 vs. 21.1, p = 0.009
Estimated blood loss (ml)	65.8 vs. 76.2, p = 0.301
*Complication (Clavien Dindo classification)*	
Grade 1 (%)	9.4 vs. 10.3, p = 0.204
≥ Grade 2 (%)	3.0 vs. 3.6, p = 0.327
Changes of eGFR (%)	15.9 vs. 16.2, p = 0.785

**Table 6:** Comparison of clinical characteristics of patients with tumor at anterior and posterior of renal hilus

Variables	Anterior vs. Posterior renal hilus tumor
n	41 vs. 74
Tumor size (cm)	2.7 vs. 3.1, p = 0.073
RENAL Nephrometry Score	8.3 vs. 8.5, p = 0.473
PADUA Nephrometry Score	10.3 vs. 10.8, p = 0.140
Surgical time (min)	119.7 vs. 120.1, p = 0.963
Warm ischemia time (min)	19.7 vs. 18.8, p = 0.559
Estimated blood loss (ml)	80.5 vs. 64, p = 0.487
Complication (Clavien Dindo classification)	
Grade 1 (%)	12.2 vs. 9.6, p = 0.192
≥Grade 2 (%)	2.4 vs. 4.1, p = 0.084
Changes of eGFR (%)	16.0 vs. 15.8, p = 0.947

We also found no significant difference in intraoperative features and postoperative outcomes when tumor larger than 4 cm was located at different positions of kidney. We compared patients whose BMI ≥ 28 and BMI < 28, with no differences in RENAL and PADUA Nephrometry Score. The surgical time was longer when BMI ≥ 28 (132.6 min vs. 122.5 min, p = 0.004). No significant differences were observed in warm ischemia time, estimated blood loss, changes in eGFR, removal time of drainage tube and catheter, postoperative feeding time, and postoperative hospital time.

## Discussion

The rRAPN is now widely used because it has advantages of direct access to the hilar, avoiding intraperitoneal structures mobilization, lower postoperative complication occurrence, shorter operative time, less blood loss, and shorter length of hospital stay. [11–15] However, rRAPN has potential disadvantages such as limited working space and higher requirements for establishing operation space. [8] In early years’ research, the transperitoneal approach is recommended for anterior or lateral tumors, [16] and the retroperitoneal approach is preferred for posterior tumors. [17] Retroperitoneal approach might be challenging for large or complex tumors. [17] However, finding in recent years’ study showed equivalent perioperative morbidity and postoperative outcomes of rRAPN and tRAPN regardless of tumor location. [18] In our study, retroperitoneal approach is proven to be an effective option for renal tumors of any location.

The retroperitoneal access has several potential disadvantages such as limited working space and unlikely to use a fourth robotic arm, especially for obese patients with a high volume of adherent perirenal fat. [8] The full flank position might be contraindicated for patients with spinal diseases. Some studies indicated that rRAPN might be challenging due to the less familiar landmarks and the technique for creating the retroperitoneal working space. [19] The surgical approach requires extensive experience in retroperitoneal laparoscopic surgeries, not only for surgeons, but also for assistants. The cases included in this study were performed by experienced surgeons in our hospital. Prior to performing rRAPNs, the surgeons had many years of experience in laparoscopic and robotic surgery, especially nephrectomies in retroperitoneal approach. In our study, we found that surgical time was longer when BMI ≥ 28 (132.6 min vs. 122.5 min, p = 0.004). However, warm ischemia time, estimated blood loss, and eGFR change 1 year after operation showed no significant difference. This result suggested that rRAPN was not a contraindication for obese patients regardless of about 10 more minutes needed for resection of perirenal fat.

Several studies confirmed the superiority of rRAPN in operative time with quicker hilar control than tRAPN. [20, 21] Warm ischemia time was shorter with rRAPN for posterior-facing tumors than tRAPN, [9] while other studies showed no significant differences between the approaches when including anterior tumors. [15, 19] A significant lower EBL was reported in rRAPN group than in tRAPN group (88 ml vs. 395 ml, p < 0.01) by Hughes-Hallet and Gin’s study. [22, 23] In our study, the mean operative time, WIT, and EBL were similar to or less than those reported in literature.

One of the most obvious potential advantages of rRAPN is the lack of bowel mobilization and low risk of bowel complications, especially for those who had abdominal surgery history. Retroperitoneal approach might be beneficial for bilateral renal tumor given that the risk of complications was higher when undergoing more abdominal surgery. In our study, 25 patients had bilateral renal tumor and underwent rRAPN twice. They all recovered well and had no abdominal complications. Thus, an obvious advantage of rRAPN is faster recovery of bowel function, which causes early postoperative feeding. Patients that had undergone rRAPN was reported to have shorter length of postoperative stay in hospital. [9, 19, 24, 25] Furthermore, we found no significant differences in drainage tube and catheter removal time, feeding time, and postoperative hospital stay of renal tumors in different locations in this study.

The positive surgical margin rate in our study was 0.45%, which was less than the reported 3.3% for cT1b renal tumors or 4.5% for cT2a renal tumors. [26] All the patients with positive surgical margins survived and did not experience tumor progression during follow-up time. A total of 50% patients for T1a renal tumor and 33% for T1b renal tumor were reported to achieve over 90% eGFR preservation. [27] Zagar’s study showed 52% of patients with renal neoplasms ≤ 4 cm preserved 90% of their preoperative eGFR. [28] In our study, the mean changes of eGFR 1 year after operation was 14.6% and approximately 39.1% of patients had eGFR > 90% of baseline. We found that for the tumor ≤ 4 cm, 50.4% of patients preserved 90% of eGFR one year after operation, which was comparable to tRAPN reported in Zagar’s study. [28] Another study showed more decreased eGFR 1 year after operation of patients that had undergone RAPN in retroperitoneal approach than transperitoneal approach (9% vs. 32% patients preserved 90% of preoperative eGFR) when tumors were larger than 4 cm. The authors gave the explaination that this result might be caused by confined surgical space in retroperitoneal approach leading to difficulties in resecting and suturing and possibility of injuring more normal renal parenchyma. [20] However, in our study, when tumor was larger than 4 cm, 29.6% patients preserved 90% of eGFR 1 year after operation in retroperitoneal approach. Our results were comparable to patients who had undergone RAPN in transperitoneal approach reported in Choi’s study and better than the results of retroperitoneal approach in his study. [20] We believed that our results might be more objective as we analysed more cases in our study. This interference suggested that large size of tumor might not be a disadvantage for retroperitoneal approach as previous studies worried. We also found no significant difference in intraoperative features and postoperative outcomes when tumor larger than 4 cm was located at different positions of kidney.

Wether rRAPN is suitable for complicated renal tumor such as endophytic and hilus tumor has not been discussed in large cohort study in the literature. In our study, there was no significant differences in surgical time between endo- and exophytic tumor group. Though there was about two more minutes on average of warm ischemia time in endophytic renal tumor, the changes of eGFR one year after surgery had no significant differences compared to patients with exophytic renal tumor. The results showed that rRAPN was suitable for endophytic renal tumors. Our study also showed no significant differences in surgical time, warm ischemia time, changes of eGFR compared anterior tumor to posterior tumor of renal hilus when tumor size and nephrometry score had no differnences. This result might suggest that rRAPN was suitable for anterior tumor of renal hilus as well as posterior renal hilus tumor.

Prognostic outcomes seemed acceptable in patients that had undergone rRAPN. In this study, mean follow-up time was 20.2 (range: 12–69) months. Moreover, 8 (0.9%) patients had recurrence, 19 (2.1%) had metastasis, and 9 (1.0%) died. One study suggested that RAPN had comparable oncological outcomes to open partial nephrectomy. [29] PN had comparable outcomes to RN in patients with larger, high-grade, and more complex tumors. [30]

Our study showed retroperitoneal approach for RAPN suits for renal tumors of all different locations as acceptable intra- and post-operative outcomes. However, it is important to make a preoperative plan to choose appropriate approach concerning surgeon’s and assistant’s proficiency of certain surgical approach, patient’s obesity level, surgical history, tumor size and location.

This study still had limitations. Although this study was the largest cohort study of patients that had undergone tRAPN reported in literature, the major limitation was its single-center retrospective design. The classification of tumor location was unspecific. A more detailed classification is needed so that we could find out more accurate indication of retroperitoneal approach of RAPN. A multicenter randomized controlled trial is needed and further long-term oncologic outcomes will be needed.

## Conclusion

Retroperitoneal approach for RAPN has confirmed acceptable intra- and postoperative outcomes and suits for renal tumors of all different locations. Large tumor size and obesity are not contraindications for rRAPN, and surgeons could choose familiar and suitable approaches that benefit the patients.

## Data Availability

All the data supporting our findings is contained in the manuscript. The datasets used and/or analysed in the current study is available from the corresponding author on reasonable request.
